# Influence of Materials Properties on Bio-Physical Features and Effectiveness of 3D-Scaffolds for Periodontal Regeneration

**DOI:** 10.3390/molecules26061643

**Published:** 2021-03-15

**Authors:** Nicola d’Avanzo, Maria Chiara Bruno, Amerigo Giudice, Antonia Mancuso, Federica De Gaetano, Maria Chiara Cristiano, Donatella Paolino, Massimo Fresta

**Affiliations:** 1 Department of Health Science, University “Magna Græcia” of Catanzaro, Campus Universitario—Germaneto, Viale Europa, I-88100 Catanzaro, Italy; nicola.davanzo@unicz.it (N.d.); mariachiara.bruno@studenti.unicz.it (M.C.B.); a.giudice@unicz.it (A.G.); antonia.mancuso@unicz.it (A.M.); 2 Department of Pharmacy, University of Chieti−Pescara “G. d’Annunzio”, I-66100 Chieti, Italy; 3 Department of Chemical, Biological, Pharmaceutical and Environmental Sciences, University of Messina, Viale Ferdinando Stagno D’Alcontres 31, I-98166 Messina, Italy; fedegaetano@unime.it; 4 Department of Experimental and Clinical Medicine, University “Magna Græcia” of Catanzaro, Campus Universitario—Germaneto, Viale Europa, I-88100 Catanzaro, Italy; mchiara.cristiano@unicz.it

**Keywords:** periodontal regeneration, 3D-scaffolds, tissue engineering, nanosystems

## Abstract

Periodontal diseases are multifactorial disorders, mainly due to severe infections and inflammation which affect the tissues (i.e., gum and dental bone) that support and surround the teeth. These pathologies are characterized by bleeding gums, pain, bad breath and, in more severe forms, can lead to the detachment of gum from teeth, causing their loss. To date it is estimated that severe periodontal diseases affect around 10% of the population worldwide thus making necessary the development of effective treatments able to both reduce the infections and inflammation in injured sites and improve the regeneration of damaged tissues. In this scenario, the use of 3D scaffolds can play a pivotal role by providing an effective platform for drugs, nanosystems, growth factors, stem cells, etc., improving the effectiveness of therapies and reducing their systemic side effects. The aim of this review is to describe the recent progress in periodontal regeneration, highlighting the influence of materials’ properties used to realize three-dimensional (3D)-scaffolds, their bio-physical characteristics and their ability to provide a biocompatible platform able to embed nanosystems.

## 1. Introduction

The presence of several species of microorganisms (commensal oral microbiota) is the main source of principal infections in the oral cavity, from moderate to severe ones [1,2,3]. Moreover, starting from the oral cavity, these microorganisms may affect the entire organism, thus spreading to other parts of the body and leading to several diseases, i.e., endocarditis, atherosclerosis, coronary artery defects and stroke [4,5].

To date, periodontal diseases affect about 20–50% of the world’s population in less serious forms and about 10% in more severe ones [6].

Periodontal affections are chronic multifactorial and immuno-inflammatory destructive pathologies which can lead to the complete loss of periodontal tissues due to the formation of pathological pocket and gingival recession [7]. Usually, the loss of periodontal tissues is a biphasic process: the first one is related to gingival inflammation without affecting the physiological state of teeth’ attachment, and its progress can be avoided or reduced by treatments with antibiotics and anti-inflammatory drugs [8]. The second stage is a chronic inflammation of the teeth supporting tissues, thus leading to their progressive detachment and bone destruction [9].

Furthermore, many risk factors such as smoking, poor oral hygiene and pathological conditions (i.e., obesity, diabetes, metabolic syndrome and osteoporosis) can be associated to periodontal pathologies, thus contributing to the damage of periodontal tissues: cementum, gingiva, periodontal ligament and alveolar bone [10,11]

According to Cardoso and co-workers [12], periodontitis can be classified as follows: necrotizing periodontitis, chronic periodontitis, aggressive periodontitis, periodontitis as a manifestation of systemic diseases, gingival diseases, abscess of the periodontium, combined periodontic-endodontic lesions and development of pathological deformities.

Patients affected by periodontitis suffer a worsening of the jaw function and the movement of the mandibular bone and, hence, of chewing, thus potentially eliciting an unsuitable nutrients’ supply to the organism [13]. For these reasons, the most used surgical practice is the implantation of prosthetic teeth [14,15] in order to restore the physiological orofacial structure and functionality.

There are various types of dental implants, made up of different biomaterials, e.g., metals, alloy (gold or nickel-chrome-vanadium), ceramics (aluminium oxide), carbon and polymers [16]. The most common implants are made up of titanium or titanium alloy [17]. These may be coated with a thin layer of hydroxyapatite or other calcium phosphate ceramics. In all of these systems, the surfaces allow a suitable bio-integration process by promoting the chemotactic recruitment of bone growth factors and, hence, the formation of a direct link between implants and bone tissues [18].

Nevertheless, the implants rejection (failure of surgical treatments) is one of the common drawbacks associated with this practice, that, in addition to the high healthcare costs and long healing times, leads to an unsuitable patients’ compliance [19].

A very suitable alternative to invasive surgical approaches may be the regenerative medicine, which can provide the potential capability to repair and regenerate various tissues involved in periodontitis, thus restoring a healthy periodontal environment [20]. This approach is based on the use of stem cells [21,22], growth factors [23,24] and biomaterials [25,26,27] or their combination in order to achieve promising treatments for periodontal regeneration [28,29].

Unfortunately, the periodontium regeneration may result in difficulty due to the presence of different kind of tissues, i.e., hard tissues (alveolar bone and tooth bone) and soft ones (periodontal membrane), as well as the direct contact of implanted materials with the mucosal cavity that may elicit the development of infections and, hence, compromise the regeneration success [30,31].

Therefore, apart from growth factors, the concomitant administration of antibacterial drugs (e.g., metronidazole, amoxicillin, amoxicillin and clavulanic acid, clindamycin) should be carried out [32,33,34]. The chronic administration of these drugs through conventional dosage forms (tablets, capsules or granules) can lead to several side effects [35], which can be overcome by using drug delivery systems suitably designed for a site-specific periodontal therapy [36,37]. Most recently, a promising approach seems to be the use of pharmaceutical nanodevices, i.e., nanoparticles and liposomes, embedded in 3D scaffolds in such a way to provide both a direct delivery of encapsulated drugs (antibiotics and/or growth factors) to injured periodontal tissues and a physical support for cells growth. This therapeutic approach can guarantee a significant improvement of the treatment efficacy by maximizing the advantages of both delivery devices [38].

In this review, we summarize the recent progress in regenerative medicine coming from the use of “smart” materials/biomaterials and nanodevices, by highlighting the importance of the substance’s features used for preparation of three-dimensional (3D) scaffolds to be proposed for the effective periodontal regeneration in the treatment of annexing diseases.

## 2. Main Scaffolds in Periodontal Tissue Regeneration

Currently, the use of scaffolds represents one of the most promising approaches in regenerative medicine, due to their ability to provide a biocompatible platform for drugs, nanosystems, growth factors and cells [28]. Indeed, these delivery systems are three-dimensional (3D) structures which may provide a physical support during the regeneration of damaged tissues [39,40,41,42]. Scaffolds must have some features to be proposed as an efficacious tool in regenerative medicine: biocompatibility with target tissues, non-immunogenicity and, where applicable, biodegradability. Moreover, to make their translation effective from bench to chairside, they must demonstrate a high reproducibility, a high stability after in vivo administration, an easy handling from health care workers and the ability to overcome the physical and mechanical stresses in the application tissue [43,44]. In particular, these scaffolds should ideally have an appropriate 3D architecture, thus resulting in a suitable “artificial extracellular matrix” which has to be able to provide a compatible support for the engraftment and the growth of cells in damaged tissues [45,46]. Scaffolds for regenerative purposes, independently from materials used for their preparation, must have a suitable porosity to allow an optimal cellular penetration and differentiation [47,48]. The crucial role of this parameter was confirmed independently by several research teams; namely, Murphy et al. [49] and Chiu et al. [50] demonstrated the importance of porous structure for scaffolds made from two different materials: collagen/glycosaminoglycan and PEG-based hydrogels, respectively. Another important parameter to be observed is the interconnection between pores thus mimicking human tissues and allowing a suitable diffusion of cellular nutrients, as well as cells’ proliferation and migration [51].

**Figure 1:**
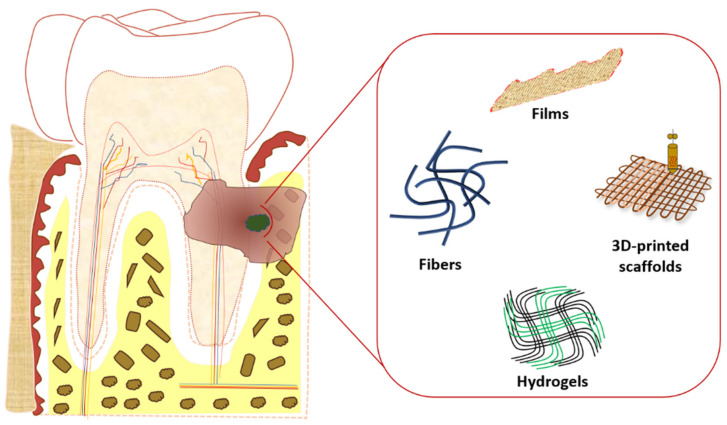
Schematic representation of the main scaffolds used in periodontitis and periodontal regeneration.

In particular, the use of suitable scaffolds for periodontal regeneration is showing an ever increasing interest by the scientific community due to the huge number of patients worldwide affected by periodontal tissues defects and the ease of their application in comparison with other body districts/tissues, e.g., heart and spinal cord [52,53]. Several scaffolds were investigated during the last two decades. Nowadays, the most studied scaffolds for periodontal application are hydrogels, films, fibers and 3D-printed scaffolds, realized using both synthetic and natural polymers (Figure 1) [54,55,56].

### 2.1. Hydrogels

Hydrogels are 3D scaffolds widely used in regenerative medicine to deliver bioactives, both in free form or encapsulated within nanodevices, as well as to provide a suitable support for cell growth in damaged tissues. The delivery of biomolecules through scaffolds can provide several advantages, i.e., a reduced degradation rate, a sustained/controlled release, thus achieving a more efficacious therapeutic approach, a reduction of their administration frequency and possible side effects [57].

Hydrogels can be realized by different methods: physical entanglement, ionic interaction and chemical crosslinking [58]. In particular, the last one is the most used because it provides more stable hydrogels which are characterized by a greater flexibility of the resulting scaffold. An important advantage of hydrogels is the possibility to prepare mucoadhesive devices by using suitable polymers, thus allowing a suitable mucosal adhesion of scaffolds with the periodontal pocket and, hence, a site-specific therapy [59,60]. Another crucial feature of hydrogels is their swelling features, which occurs when in contact with aqueous solution as a consequence of their polymeric network. These features have been widely exploited in periodontal diseases because they allow scaffolds to adapt themselves to injured periodontal tissues following administration [61]. In order to significantly improve the adaptability to periodontal pocket [52], thermosensitive hydrogels with in situ-crosslink ability were prepared by exploiting their sol-gel transition temperature [62], thus achieving injection ease into periodontal being liquid at room temperature and gel at body temperature following administration. In these attempts, Pakzad et al. [63] combined chitosan/β-glycerophosphate and gelatine in order to realize a thermosensitive hydrogel for a suitable periodontal application. The addition of gelatine at 0.5% *w/v* to chitosan/β-glycerophosphate hydrogel decrease its gelling time and gelling temperature from 35 °C at 33 °C, thus improving the scaffold strength at 37 °C. It was demonstrated that the improved in situ gelling properties of this hybrid hydrogel provided a more suitable controlled and sustained release of encapsulated metronidazole by reducing its release from 85% (hydrogel realized without gelatine) to 76% after 1 week. This hydrogel was in vitro tested and a suitable antimicrobial activity against Clostridium sporogenes bacteria was observed. The cytotoxicity evaluation on human embryonic kidney cells demonstrated a reduced cell viability when cells were incubated with hydrogel extractive solution having a metronidazole concentration ranging from 500 to 1000 µM. Conversely, no significant cytotoxic effect was observed for metronidazole concentration ranging from 5 to 50 µM, thus evidencing a dose-dependent cytotoxic effect related to the drug concentration and not to hydrogel composition and, hence, emphasizing hydrogel potential application for in vivo periodontal affections [63]. The pivotal role of hydrogel thermoreversibility in periodontal application was also highlighted [64] by preparing chitosan/β-glycerophosphate thermoresponsive hydrogel embedding bone-morphogenetic protein 2 plasmid DNA-loaded chitosan nanoparticles. This “composite hydrogel” showed a suitable in situ gel-formation feature following administration both in rats and beagle dogs and a significant endogenous bone regeneration was obtained in both animal models after 8 weeks from implantation. Therefore, this composite hydrogel provided at the same time an optimal gene delivery and a suitable physical support for the recruitment of cells directly from the target tissue, thus resulting in an effective scaffold for endogenous periodontal bone regeneration [64]. The embedding of nanoparticles into hydrogels also overcame one of the main disadvantages of these scaffolds, i.e., their incapability to deliver hydrophobic compounds due to their great aqueous content [58].

Another challenge in the periodontal application of hydrogels is their sterilization, which could compromise the 3D architecture by destroying the polymeric network and, hence, result in an unstable and inefficacious scaffold [65]. In order to fulfil this requirement, Miranda et al. [66] prepared a chitosan-hyaluronic acid hydrogel scaffold by chemically modifying both polymers in such a way to promote a crosslinking reaction to form a more stable hybrid hydrogel, which could be submitted to a sterilization process. The synthesized scaffold showed a proper porosity, suitable in vitro swelling features and no-significant morphological change following sterilization through UV and gamma irradiations, although the latest one altered hydrogel swelling capability. This hybrid scaffold was also in vitro tested and a greater cellular activity as well as a CD44 protein production of cells seeded on it (osteoblast-like cells (MG63) and Swiss albino mouse embryo tissue (NIH3T3) fibroblasts) were observed in comparison with hybrid scaffolds made up of a single polymer. These results evidenced an improvement of cellular migration rate on hybrid hydrogels made up of both polymers, thus prompting their potential use for periodontal regeneration [66].

Similarly, chitosan was used to prepare a chitosan/β-sodium glycerophosphate/gelatine thermosensitive hydrogel [67]. In this investigation, aspirin and erythropoietin (EPO) (an anti-inflammatory drug and a growth factor, respectively) were loaded into the scaffold. In vitro experiments of drugs’ release showed a cumulative release after 3 days of 86.6% and 69.4% for acetylsalicylic acid and erythropoietin, respectively. Authors speculated that the greater release rate of the anti-inflammatory drug was due to its smaller molecular weight than the growth factor, by highlighting the advantage of an initial massive release of the anti-inflammatory drug during the early stage of periodontitis treatment. Both drugs showed a bi-phasic release profile up to 21 days, showing the acetylsalicylic acid and growth factor release of 94.2% and 83.4%, respectively, after 8 days of incubation, and then the potential release of small remained drugs up to the end of the study (from day 9 to day 21). The investigated hydrogel did not show significant toxicity both in vitro and in vivo up to 21 days. Moreover, an in vivo study on rats and histological observations demonstrated that dual drug-loaded hydrogel provided at the same time a great anti-inflammatory effect and a significant alveolar bone regeneration, thus suggesting the crucial role of localized anti-inflammatory therapy and the ability to improve the periodontal regeneration following association with growth factors [67].

The increased stability of aspirin due to its inclusion within hydrogel was also confirmed by Zhang et al. [68]. Namely, in this investigation a PEG-based hydrogel was prepared and the osteogenic-inductive activity of acetyl salicylic acid (ASA) on periodontal ligament stem cells was studied in vitro by refining the most suitable drug concentration at 100 µg/mL, that was used throughout further experiments. A biphasic drug release rate from this hydrogel was observed: a first phase characterized by a 80% release of the encapsulated drug up to 8 days, followed by a plateau-phase up to 14 days. In vitro osteogenic experiments demonstrated the formation of calcified nodules after 14 days of cells exposition to ASA-loaded hydrogel as well as an increased expression of osteogenic markers, i.e., RUNX2, ALP and OCN, starting from the 7th day. In vivo experiments on mice-bearing periodontal bone defects confirmed the in vitro ones, that is, a greater regenerative capability of hydrogels containing both stem cells and ASA than hydrogels with only stem cells was observed [68].

### 2.2. Films

Films are thin 3D scaffolds made up of a polymeric matrix which may contain one or several bioactive substances to be released in target tissue by a diffusion mechanism and/or matrix dissolution or erosion [69]. The size and the shape of this scaffold can be appropriately engineered in order to be adapted to the specific damaged periodontal site. The possibility to control these parameters during their preparation can allow their easy insertion into injured sites and in some cases an easier handling than hydrogels was demonstrated [70,71]. The techniques mainly used for film preparation are the polymer solution and the polymer melt. The polymer solution is based on the use of organic or inorganic solvents which must finally be removed, while in case of polymer melt polymeric materials are heated to make it fluid [72].

Several investigations described the use of films containing different bioactives for the treatment of periodontitis and for their application in periodontal injuries. In particular, chitosan films containing two antibiotics drugs, i.e., metronidazole and levofloxacin, were prepared as therapeutic periodontal intra-pocket biodegradable film for the sustained and controlled release of payloads. Interestingly, the influence of concentrations of materials used for film preparation, i.e., chitosan, glutaraldehyde (crosslinking agent) and propylene glycol (plasticizer), on final physicochemical features of films was investigated, thus evidencing that the drugs’ release rate was mainly affected by the presence of crosslinking agents. Indeed, the absence of glutaraldehyde elicited a drugs’ release of 80% during the first 24 h of incubation, while the remaining amount was released in the following 4 days. Films prepared using glutaraldehyde were characterized by a more suitable sustained drug release profile: a 60% release of loaded drugs during the first 24 h and a continuous release of the remaining amount up to 7 days. The amount of chitosan and glutaraldehyde positively influenced the T_90_ value (time required to release the 90% of entrapped drugs), i.e., the greater the amount of the two film components the greater the T_90_ value, while there was an inverse correlation between the T_90_ value and the plasticizer amount, probably due to the alteration of film hydrophilicity. The scaffold with the more suitable feature for periodontal applications was made up of 0.8 g of chitosan, a low amount of plasticizer (0.16 g) and a largest amount of glutaraldehyde (0.04 g), thus being selected for further investigations. In vitro antibacterial activity of this therapeutic film was evaluated against *E. coli* and *S. aureus* and compared with film containing only levofloxacin, using the unloaded film as negative control. Dual drug-loaded films showed a more efficacious antimicrobial activity than scaffolds loaded with only levofloxacin, especially in case of long incubation times (up to 7 days). Owing to these promising results, authors designed a clinical study in order to evaluate the efficacy of this therapeutic film as an adjuvant treatment of traditional scaling and root planing (SRP) procedures. In particular, it was demonstrated a suitable patients’ compliance regarding to investigated films throughout the study (8 weeks) and a greater decrease of periodontitis’ clinical markers in case of SRP associated with two drugs-loaded film than SRP alone or the combination SRP/film containing only levofloxacin [73].

Sparfloxacin-loaded chitosan-based film was also proposed as an antimicrobial device for the treatment of periodontal diseases [74]. In this case, the film preparation was carried out using chitosan alone or in combination with various co-polymers, i.e., hydroxypropyl cellulose, carboxymethyl cellulose and Eudragit RL100. The presence of co-polymers significantly improved the film tensile strength, thus achieving the most suitable scaffold with a co-polymers concentration of 30% (*w*/*w*). The drug was uniformly loaded into the film formulation at a concentration of 0.5 mg/12 mm^2^ and the antimicrobial activity was in vitro tested on *Staphylococcus aureus* by demonstrating a suitable anti-bacterial activity up to 96 h. Also in this case, drug release experiments showed a biphasic release profile, i.e., an initial burst release during the first 24 h, followed by a sustained and continuous release up to 8 days, through a diffusion mechanism. Moreover, long-term stability studies demonstrated no change of the main film features up to three months. Another investigation on chitosan-based films for in situ periodontal application was carried out by Ahmed and co-workers [75] with the aim of entrapping tetracycline antibiotic and achieving a “low-dose delivery systems.” Some films were prepared without cross-linking agent (glutaraldehyde) and contained different amount of the drug, i.e., 10%, 20% and 30% *w*/*w* to the weight of polymer, while other films were prepared using glutaraldehyde (2% *w*/*w*) and the drug at a concentration of 30% *w*/*w*. Glutaraldehyde was added to improve the matrix network and then to achieve a sustained and suitably controlled drug release. Indeed, cross-linked films showed an in vitro sustained release up to 21 days with respect to uncross-linked ones characterized by a 7 days’ release. A tensile strength increase was also observed as a function of the drug amount increase, showing a minimum value for drug-free films. Conversely, the elongation percentage decreased by increasing both the drug amount and the cross-linking reaction time. Also in this case, a suitable film stability was observed up to 10 weeks. The in vitro antimicrobial activity of therapeutic films was evaluated on *Streptococcus mutans* and a suitable antibacterial activity was observed for all drug-loaded films, while a controversial finding comes out of this investigation because no antimicrobial activity was observed for drug-free film [75] despite chitosan having per se a well-known antimicrobial activity [76].

Similar approaches were also investigated by different research teams, which proposed the inclusion of various drugs, both synthetic and natural, i.e., tenoxicam, moxifloxacin, ciprofloxacin, metformin and curcumin, into polymeric film for periodontitis treatment [77,78,79,80,81].

### 2.3. Fibers

Fibers are another drug delivery system that can contain various payloads with a promising applicability in periodontal regeneration. Due to their 3D structure, fibers can show different architectures, thus having the possibility to achieve various types of devices to be applied directly onto the periodontal tissues or formulated as micro-tablets or micro-capsules [69]. Among the techniques used to prepare these scaffolds, the most common one is the electrospinning due to its low costs, easy applicability and high reproducibility [82], as well as the possibility of obtaining fibers that have advantages over those obtained with traditional methods, i.e., accurate sizes, greater flexibility and suitable mechanical features [83,84]. Indeed, the appropriate setting of electrospinning parameters during the fiber production, i.e., voltage and polymer solution viscosity, can allow the modulation of various fiber features and, hence, of the resulting scaffold, i.e., ability to provide a physical support for drugs, growth factors and cells, such as porosity and surface area. Bio-actives can be realized from fibers through three mechanisms: desorption from fibers’ surface, solid-state diffusion from fibers and in vivo fiber degradation [85].

As above described, the chronic administration of anti-inflammatory drugs using conventional formulations, i.e., tablets or capsules during periodontal diseases, may elicit some side effects such as gastrointestinal irritation, hepatic and renal injuries. Therefore, the use of fibers as drug delivery systems can mitigate these drawbacks, thus providing a controlled and localized release of payload in periodontal pocket [86,87]. In particular, piroxicam-loaded electrospun fibers’ mats were prepared for periodontal application and their features were compared with bio-film loaded with the same drug. Both scaffolds were realized using the same polymers: chitosan, poly-(vinyl alcohol) and hydroxyapatite. The synthesized nano-fibers appeared thin, smooth and overlapped, thus providing a thick network with an even distribution of loaded drug. Conversely, bio-films showed a worst distribution of piroxicam as drug aggregate and crystal on their surface were observed. As a consequence, a more suitably controlled and sustained drug release was observed from fibers than from films, which showed a massive initial release. In any case, in vitro biocompatibility tests demonstrated that both scaffolds provided a suitable physical support for VERO cell line growth up to 72 h without compromising the cells’ morphology. The best results were obtained by using nano-fibers due to their greater porosity and surface area, thus further confirming the crucial role of these parameters to achieve both a suitable reservoir system and physical support for cell growth [88].

The pivotal role of these two parameters was also confirmed by Lofti et al. [89], who compared two bilayered scaffolds with the same chitosan-based film but having two different coatings. The collagen coating surface was realized by two different approaches: electrospunning and casting one. It was demonstrated a greater metabolic activity of rabbit mesenchymal stem cells seeded on bilayered scaffold with fibrous collagen coating compared with scaffolds made up of only chitosan or with solid wall collagen coating, as well as the greater surface area of scaffold with nanofibrous collagen coating improved the osteogenic differentiation of investigated cells, especially between the 14th day and the 21st day. In vivo tests were in very good agreement with in vitro ones, namely, periodontal bone injury-bearing rabbits treated with fibrous collagen/chitosan-based bilayered scaffolds showed a more homogeneous and extensive bone regeneration compared with others scaffold and no significant inflammation reaction in the application site, thus evidencing a suitable biocompatibility [89].

Similarly to film-based scaffolds, the combination of two or more materials improves the performance of fiber-based scaffolds. Indeed, nanofiber-based scaffolds prepared by electrospinning and made up of a combination of calcium alginate and poly-(lactid acid) (PLA) showed more effective performance for periodontal applications than those prepared with neat PLA fibers [90]. Namely, SEM analysis evidenced fibers with a uniform mesh size in case of PLA/calcium alginate. The addition of calcium alginate improved the physicochemical properties of nanofibers by increasing their elastic modulus, tensile strength and elongation. Calcium alginate mitigated the hydrophobicity of PLA, thus improving the wettability index and, hence, providing a suitable support for cell growth. The improved physicochemical features of this nanofiber-based scaffolds were in agreement with in vitro findings, which have demonstrated a suitable cell viability and adhesion of bone marrow stromal (BMS) cells and periodontal ligament cells (hPDLCs) on calcium alginate/PLA fibers. Furthermore, BMS cells seeded on these fibers showed the presence of a mineralized matrix with an improved expression of bone mineralization genes, i.e., Runx-2, OPG, Collagen I and RANKL, thus suggesting that this scaffold may enhance osteogenic properties. Unfortunately, despite these encouraging results, cells seeded on these fibers showed an increase in the expression of inflammation mediators. This drawback may compromise the in vivo application of alginate/PLA nanofiber-based scaffold, thus requiring further approaches, such as the block of TLR4 expression, to mitigate inflammation response [90].

### 2.4. 3D-Printed Scaffolds

The 3D-printed scaffolds are probably the most promising ones in periodontal tissue regeneration, to date. The main advantage of this procedure is the possibility to set accurately the parameters during the production phases, thus allowing the realization of a physical support with a 3D architecture similar to the extracellular matrix in physiological conditions [91]. Indeed, this technique can be managed by using a suitable software, which allows a fine control of several parameters during the production [92,93], and it is (to date) the only one able to ensure an effective scaffold “multiphasic” configuration that reflects the complexity of periodontal tissues [94,95]. The 3D printing technique can strongly reduce the time and costs of scaffolds production, thus improving at the same time their reproducibility. These features can speed up the scaffold translation from bench to clinic [56,96]. In these attempts, a 3D-printed hybrid scaffold, mimicking the interface between the periodontal ligaments and periodontal bone, was realized for periodontal ligament regeneration to restore the type-I collagen bundles/periodontal ligament fibers [97]. Briefly, human periodontal ligament cells were extracted from molar and premolar teeth of patients and seeded on polycaprolactone-based 3D printed scaffold. These scaffolds were in vivo implanted on rats in order to evaluate their regenerative capability and the formation of de novo mineralized tissue. In vivo tests demonstrated that periodontal ligament cells on implanted scaffold led to the formation of type I collagen fibers and to a suitable alignment of bone-ligament complexes, thus ensuring the proper orientation and 3D architecture of multiple periodontal tissues [97].

A further evidence of the advantages of “multiphasic” scaffolds for periodontal application was the regeneration of periodontal complex tissues using a tri-phasic poly-(caprolactone)/hydroxyapatite 3D-printed scaffold [98]. The constituents of these scaffolds are phase A designed for cementum/dentine interface (100 μm microchannels), phase B designed for periodontal ligaments (600 μm microchannels) and phase C designed for alveolar bone (300 μm microchannels). The tri-phase 3D-printed scaffolds were loaded with amelogenin (a growth factor for cementum and bone regeneration), a connective tissue growth factor to stimulate periodontal ligament regeneration and a bone morphogenetic protein 2 as osteoconductive factor to stimulate alveolar bone regeneration. The aim of loading three different growth factors was the achievement of a simultaneous regeneration of different periodontal tissues. Moreover, the three growth factors were encapsulated into poly(lactic-co-(glycolic acid)) microspheres and, hence, they were embedded into poly(caprolactone)/hydroxyapatite 3D scaffold to improve their stability. The so-realized multistage scaffolds were tested in vitro according to the following procedure: dental pulp stem/progenitor cells (DPSCs), periodontal ligament stem/progenitor cells (PDLSCs) and alveolar bone stem/progenitor cells (ABSCs) were extracted from patients, embedded in a collagen solution and then seeded on scaffold phase A, B and C, respectively. Results demonstrated the formation of a dense and polarized mineralized tissue in phase A, collagen-like fibers in a no-mineralized matrix in phase B and a mineralized tissue in phase C. These findings were also supported by in vivo experiments following the implantation of tri-phase multistage 3D-printed scaffold in immunodeficient mice. In particular, only DPSCs were seeded on scaffolds before their in vivo application and the regeneration of multiphasic tissues mimicking the periodontal complex tissue, i.e., formation of new dentin/cementum-like structures with a periodontal ligament-like tissue interconnected between them, was observed after 4 weeks from implantation [98]. Therefore, these encouraging results prompt the use of 3D-printed scaffolds to drive a simultaneous regeneration of different periodontal tissues.

## 3. Main Materials for Periodontal Scaffolds Manufacturing

**Figure 2:**
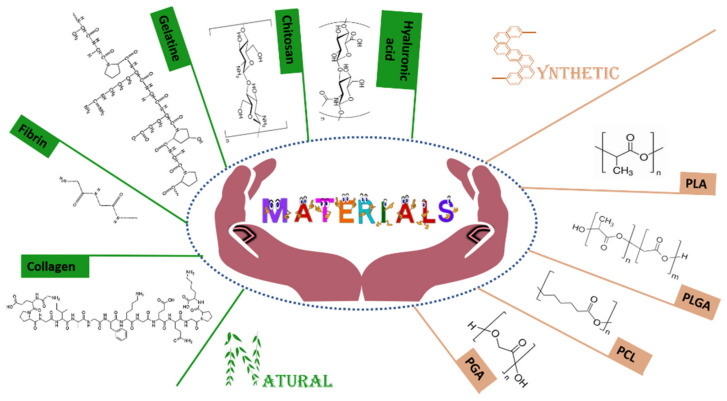
Natural and synthetic polymers mainly used in periodontal tissue regeneration.

To date, several polymers, both natural and synthetic, have been investigated to real-ize 3D scaffold for periodontal regeneration (Figure 2). These constitutive materials strongly affect the features of resulting scaffolds and are chosen as a function of their physicochemical characteristics and the application site. In fact, 3D scaffolds should adapt themselves in the application site, resist the physicochemical and biological stresses and, hence, provide a suitable support for the regeneration of injured periodontal tissues [94].

### 3.1. The Main Synthetic Polymers

The term “synthetic” is used for polymers synthesizes in the laboratory through several processes, i.e., polymerization, crosslinking, etc. [99]. The main advantages of these polymers are the lower cost of production than natural ones and the possibility to modify their structure to fulfil the therapeutic requirements, i.e., in vivo degradation rate, mechanical strength, etc. [100,101]. Synthetic polymer-based scaffolds can be prepared on a large scale by sharply controlled processes, thus ensuring a great reproducibility between batches [101].

The principal disadvantages of synthetic polymers are related to their reduced biocompatibility in comparison with natural ones, thus being a less suitable support for cell adhesion and/or migration, hence, leading to a poor regeneration efficiency of damaged periodontal tissues. In order to mitigate these drawbacks, synthetic polymers are often used in association with natural ones [102]. The potential adverse reactions elicited by the in vivo degradation products of this class of polymers may be another problem to be taken into account [103,104,105].

Currently, the main synthetic polymers used in periodontal tissues regeneration are polyester, i.e., poly(glycolic acid) and poly(lactic acid), their copolymer poly(lactic-co-(glycolic acid)) and polycaprolactone.

#### 3.1.1. Poly(glycolic Acid)

The poly(glycolic acid) or PGA is a biocompatible, biodegradable and non-toxic aliphatic polyester widely used for several biomedical applications. This polymer shows a suitable mechanical strength and then it may be a suitable biomaterial for 3D scaffold preparation [101]. In these attempts, a PGA-based fiber network was prepared [106] to obtain a physical support for adhesion and proliferation of human periodontal ligament (hPDL) stem cells, which showed after 7 days from their seeding a suitable cell growth and extracellular matrix production, a greater expression of collagen type I, collagen type III and fibronectin mRNA levels than those observed in two-dimensional cultured cells. hPDL stem cells-loaded scaffolds were then subcutaneously implanted in BALB/c-nude mice, which were sacrificed at different time points (2, 4, 6 and 8 weeks) and a suitable vascularization of implanted construct as well as the formation of hPDL stem cell-derived new tissue were observed. Based on these results, the authors [106] claimed the potential use of PGA-based fibers as a scaffold for the delivery of hPDL stem cells to be applied for the treatment of periodontal regeneration.

PGA was used to realize a “membrane scaffold” [107]. Its ability to provide a suitable support for periodontal regeneration was investigated and compared to collagen-based scaffold with a similar morphology. Periodontal ligament fibroblast spheroids were used as cellular models and similar results were obtained for both scaffolds in terms of cell adhesion, differentiation, migration and new extracellular matrix production. In particular, Alamar blue assay showed the maximum cell growth rate at the 20th day, while the extracellular protein expression, i.e., collagen type I, periostin and runx2, became noticeable in both membranes at the 15th day of incubation. The scanning electron microscopy was used to evaluate the cell migration rate and a round shape of investigated cells and their wrapping growth around the PGA fibers for the entire incubation time was observed. After 20 days of incubation, PGA-based membrane demonstrated an initial degradation. These findings demonstrated the ability of PGA to provide a suitable artificial microenvironment for periodontal regeneration by ensuring an appropriate cell proliferation, adhesion and migration, similar to those observed for scaffold made up of collagen, which is an extracellular matrix element [107].

#### 3.1.2. Poly(lactide Acid)

The poly(lactide acid) (PLA) is a biodegradable aliphatic polyester with a greater hydrophobicity than PGA and it is in vivo metabolized in lactic acid, which is a product of physiological carbohydrate metabolism [108]. It can show two different optical isoforms, i.e., poly(d-lactic acid) (PDLA) and poly(l-lactide) (PLLA) [27,109]. An interesting use of this polymer was the synthesis of PLLA/chitosan electrospun fibers [110] in order to realize a composite membrane for periodontal application. In this study, chitosan was used to reduce the hydrophobicity of PLLA and, hence, to increase cells’ attachment with the aim to have a more suitable physical support for periodontal regeneration. Interestingly, the use of chitosan as co-polymer increased the in vitro scaffold degradation rate of 20% in 6 weeks, with respect to fibres made up of only PLLA. These results suggested that the presence of chitosan may reduce the potential side effects linked to the high hydrophobicity and the low degradation rate of PLLA scaffolds, thus evidencing the real possibility to combine natural and synthetic materials to modulate the final feature of potentially safe scaffolds [110].

PLLA and PGA are usually used in form of copolymer poly(lactic-co-(glycolic acid)) to improve the physicochemical features of scaffold to be used in tissue regeneration

#### 3.1.3. Poly-[Lactic-co-(Glycolic Acid)]

The poly-[lactic-co-(glycolic acid)] (PLGA) is a linear aliphatic copolymer synthesized by the association of PLA and PGA through ester bond. During the polymerization processes, these two co-monomers can be associated in different molar ratio and allotropic forms (i.e., crystalline or amorphous one), thus showing distinct physicochemical features of resulting scaffolds, e.g., a lower mechanical strength of scaffolds realized with the amorphous form with respect to the crystalline one. This polymer is biocompatible and biodegradable, and several studies were carried out for its application in periodontal regeneration [111].

A fibronectin-functionalized electrospun PLGA scaffold was realized [112] to improve the periodontal ligament cells adhesion to this physical support. In vitro studies demonstrated a greater adhesion rate of investigated cells to the fibronectin-functionalized scaffold with respect to no functionalized ones. Moreover, fibronectin allowed a more homogeneous cell attachment on the entire scaffold surface, thus showing a novel extracellular matrix deposition on its and an acquired migration ability of seeded cells. Therefore, fibronectin-functionalized PLGA fibers are a more suitable scaffold for periodontal regeneration than non-functionalized ones, because they better mimic the physiological features [112].

PLGA was also investigated to realize hydrogels for the chronic treatment of periodontitis. An injectable scaffold made up of PLGA and hydroxypropyl methylcellulose loaded with chlorhexidine, an antiseptic drug, was realized [113], and its syringeability, texture profile and swelling/shrinkage properties were compared with two gels already on the market: Parocline^®^ (minocycline gel) and Chlo-site^®^ (chlorhexidine gel). The investigated novel hydrogel demonstrated a more suitable syringeability than Parocline^®^ and Chlo-site^®^, while the texture parameters, i.e., hardness, springiness and resilience, were halfway between the two gels on the market. These results demonstrated more suitable physicochemical properties of PLGA/hydroxypropyl methylcellulose hydrogel for an easier periodontal application. The presence of HPMC played a crucial role because it was able to improve gel property, thus providing a more stable and suitable physical support for a potential in vivo periodontal application [113].

Three years later, the same research team investigated the possible co-delivery through the same hydrogel both chlorhexidine and ibuprofen. The antimicrobial effect of resulting therapeutic hydrogel was in vitro investigated on several periodontal microbial strains, i.e., *Streptococcus sanguinis, Streptococcus constellatus, Actinomyces odontolyticus, Veillonella dispar, Fusobacterium nucleatum, Fusobacterium naviforme, Prevotella nigrescens* and *Porphyromonas gingivalis*. In this study, the authors investigated several drugs ratio (1.5:1.5; 5.3:5.3; 16.1:16.1 %/% *w*/*w*) showing the best antimicrobial effects of the two latter concentrations. Indeed, the hydrogel containing both drugs at the lowest molar ratio concentration did not demonstrate any significant advantages in comparison with marketed chlorhexidine dichloride gel (1% *w*/*w*) and chlorhexidine digluconate gel (0.5%). Conversely, the two hydrogels with the highest drug concentration demonstrated a suitable antibacterial effect on above mentioned microbial cells after 24 h of incubation, thus demonstrating the synergistic effect of the co-loaded drugs. Moreover, the encapsulation of both drugs in all investigated concentrations did not compromise the texture and syringeability profiles of therapeutic scaffolds, suggesting their potential implication in periodontitis [114]. 

Another fascinating approach was proposed by Reis et al. [115] through the synthesis of multilayered scaffold made up by PLGA and calcium phosphate. This scaffold showed a high stability and adaptability to the application site, with a suitable interconnected macroporosity that allowed the blood reabsorption after implantation. The aim of this study was to develop a bilayered construct for a proper in vivo regeneration of functional periodontal tissues. This scaffold was tested on Class II furcation defects in dogs for 30, 60, 90 and 120 days. The histological analysis showed a suitable regeneration of the main periodontal tissues i.e., cementum, alveolar bone and periodontal ligament on treated animals. Indeed, the rigidity of this construct strongly reduced its collapse in comparison with absorbable membrane leading to a proper physical support for the regeneration of damaged periodontal tissue [115]. 

#### 3.1.4. Polycaprolactone

The polycaprolactone or PCL is a linear aliphatic polyester, biodegradable and biocompatible towards various tissues of the body, their mechanical properties fit with the application in scaffolds’ synthesis. It is synthetized by a ring opening polymerization of ɛ-caprolactone and shows a low water solubility and slow degradation rate in caproic acid by ester bond hydrolysis [116].

Recently, Batool et al. [117] investigated the use of this polymer through the synthesis of electrospun polycaprolactone scaffold for periodontal application in order to realize a reservoir system to site-specific deliver ibuprofen in periodontal pocket. The resulting drug-loaded membrane showed a high in vitro efficacy, strongly reducing the migration and proliferation rate of epithelial cells and fibroblasts exposed to *Porphyromonas gingivalis* lipopolysaccharide (*Pg*-LPS), in order to mimic the in vivo inflammation environment. These data were in vivo confirmed in a periodontitis mouse model after 22 days of implantation. Indeed, ibuprofene-loaded membrane strongly enhanced the wound healing, reducing at the same time the inflammation-mediated bone destruction. These results emphasize the synergistic effect of anti-inflammatory drugs and PCL-based 3D scaffold, providing a localized therapy able to manage the periodontal inflammation process and promote the regeneration of injured tissues [117].

Similarly to other investigated polymers, PCL is also usually associated with several materials (both natural or synthetic) to improve the features of resulting scaffolds. In these attempts, Dan et al. [118] combined PCL to calcium phosphate (CP), an osteoconductive material, in order to provide a construct to enhance the regenerative properties of patients cells’ sheets derived from different periodontal tissues, on an athymic defect in a rat model. In particular, Alveolar bone (AB), gingival margin (GM) and Periodontal ligament (PDL) cell sheets were obtained starting from the proper primary cells and then implanted in rats by the use of physical support of PCL/CP scaffold. The addition of this construct of AB and PDL cell sheets led to a significant periodontal tissues engraftment, especially after 4 weeks from implantation. Opposite results were obtained with the use of GM cell sheets. Therefore, in this study the authors demonstrated that the combination of osteoinductive properties of this scaffold and exogenous AB/PDL cell sheets may provide affective regenerative approach in periodontal tissues [118]. 

The ability of CP to improve the regenerative properties of PCL-based scaffold was further demonstrated by Costa et al. through the synthesis of biphasic construct. The improved regenerative properties of CP-coated scaffold compared to no-coated one were in vitro demonstrated by the use of sheep-derived osteoblast and periodontal ligament cells up to 6 weeks. The cells-loaded scaffolds were then ectopically subcutaneous implanted on rats and the new bone formation was monitored up to 8 weeks. In these attempts, the best results were displayed by cells-loaded multi-layered scaffolds that were previously cultured in osteogenic medium, also displaying a new vascularization of the entire construct, through histological analysis. Furthermore, in response to the high porosity of CP-coated construct, the scaffold also promoted the functional cells engraftment showing the formation of new periodontal ligament fibers [119].

PCL nanofibers were also investigated for the delivery of resveratrol, a natural compound characterized by anti-oxidative and anti-inflammatory effects, in order to provide a therapeutic scaffold for a potential application in periodontitis [120]. In this study, it was demonstrated that PCL fibers were able to retain resveratrol due to the formation of hydrogen bonds, as confirmed by FT-IR spectroscopy. Moreover, the authors showed a correlation between the release profiles of payload from the therapeutic scaffold and the fibers’ synthesis conditions, i.e., drug amount and solvent mixture. In these attempts, the best results were obtained using the mixture chloroform: acetone (3:1 volume ratio) without the adding of sodium iodine. Indeed, these conditions provided a high inclusion of resveratrol in the scaffold’s matrix, then resulting in a better controlled kinetic release. The release ratio was also affected by the amount of drug used during the preparation. Indeed, when a 5% *w/w* ratio of resveratrol was added (using the mixture chloroform:acetone 3:1), the 80% of payload was retained after 12 h. Conversely, when the fibers were realized using 20% *w*/*w* of drugs in the same conditions, only the 20% was retained after 12 h due to the rapid release of drug crystals adsorbed on the surface of fibers, then resulting in the same absolute amount of resveratrol retained after this incubation time, independently from the loaded drugs during the preparation stages (5% or 20%). Therefore, this study demonstrated that the use of PCL fibers provides a resveratrol-reservoir system and the possibility to modulate its features through the proper setting of production parameters [120].

Moreover, the kinetic release profile of payload may be further modified when two or more drugs are delivered by the same scaffold, in response to the established interaction among the loaded bioactives. In these attempts, Dias et al. demonstrated a slower release of oxytetracycline hydrochloride (OTC) when it was co-loaded with zinc oxide in PCL nanofibers, probably due to the electrostatic interaction between the two proposed drugs [121]. In fact, the OTC release was about 100% vs. 31.5% after 10 h when it was included in nanofibers without or with zinc oxide, respectively. The obtained co-loaded nanofibers showed no significant cytotoxic effects on mouse L929 fibroblast cell line up to 48h, demonstrating a suitable antimicrobial effect against the main anaerobic bacteria involved in periodontitis, in vitro. Based on these results, the authors highlighted that OTC/zinc oxide co-loaded PCL nanofibers showed a potential application in periodontal infections [121].

As above described, the association of synthetic and natural materials may improve the features of resulting scaffold leading to a physical support that better mimics the periodontal environment, then enhancing its regenerative capabilities [122]. In these attempts, PCL nanofibers were combined with porous gelatine in order to provide a physiological-like architecture for periodontal ligaments regeneration, improving at the same time the bone-contact biocompatibility of resulting scaffold. The importance of PCL fibers alignment was investigated by the use of random electrospun PCL fibers or aligned ones, in order to evaluate the influence of this characteristic in the regeneration of new functional periodontal tissue. The crucial role of this parameter was in vitro confirmed, showing an elongated shape and a proper morphology of human periodontal ligament mesenchymal cells (PDLSCs) seeded on aligned fibers compared to the ones cultured on random fibers that exhibited an irregular and spread morphology. In vivo analysis demonstrated a similar trend, showing the formation of random ligament tissue after 3 weeks from the implantation of “random scaffold” and a more oriented ones for “aligned construct.” Similar results were observed after 6 weeks, showing the greater formation of new bone tissue in the aligned scaffold compared to random one. Moreover, the multilayered structures did not cause any significant inflammation reaction, because of the presence of gelatine [123].

Therefore, these data confirmed the pivotal role of two main essential parameters during the synthesis of periodontal scaffolds: a proper and well-controlled architecture in order to provide a physical support able to guide the regeneration of functional tissues, and the use of natural materials (also in association with synthetic ones) to better mimic the physiological conditions after implantation.

### 3.2. The Main Natural Materials 

Natural materials used to realize scaffolds can be obtained from different renewable sources, such as plants, animals and microorganisms [124]. The main natural materials used for this purpose are hyaluronic acid, collagen, gelatine and chitosan, even if recently fibrin and its application in tissue regeneration have been gaining attention from the scientific community [125,126].

These natural materials show several advantages, mainly that they are biocompatible towards many tissues of the body and are biodegradable compared to synthetic ones. Moreover, natural polymers show a high elasticity, hydrophilicity and moldability, making them suitable materials for the scaffolds’ synthesis [127]. On the contrary, some unfavorable features could reduce their applicability in clinical settings, such as their ability to activate immune response, their poor mechanical properties, the hardness of purification processes and batch-to-batch variability [128]. 

Based on these observations, a suitable approach could be represented by the combination of natural materials with synthetic ones, in order to obtain a “hybrid scaffold” that maximizes the advantages of both polymers.

In the following paragraphs, we describe in detail the characteristics of the main natural materials used for the construction of periodontal scaffolds, and the scientific efforts made to improve their performance.

#### 3.2.1. Hyaluronic Acid

Hyaluronic acid, also known as hyaluronan or hyaluronate, is a natural component of extracellular matrix and one of the most investigated biomaterials for tissue regeneration. It is a natural glycosaminoglycan with repeating units of d-glucuronic acid and N-acetyl-d-glucosamine present in connective tissue, synovial fluid and other tissues. In clinical practice, it is widely used for its multiple pharmacological activities such as anti-inflammatory, anti-edematous and anti-bacterial effects [129].

Regarding tissue regeneration, the mainly exploited properties of hyaluronic acid are its suitable viscoelastic characteristics and its ability to manage osmotic pressure and lubrification processes in the application site. These features, in addition to its contribution in cell growth and differentiation, in both mineralized and no-mineralized tissues, make this polymer an excellent material for the synthesis of periodontal scaffolds [130,131,132]. 

Despite its relatively new application in periodontal affections, recently, a clinical trial carried out by Pilloni et al. [133] evaluated the adjuvant effects of hyaluronic acid in the coronally advanced flap surgical technique for gingival recession therapy, in order to enhance the effectiveness of this practice. In particular, the clinical trial was achieved in 18 months on 30 patients, divided in two groups: the first group (*n* = 15 patients) was treated with hyaluronic acid in combination with coronally advanced flap, while the second group (*n* = 15 patients), considered as control group, was only treated with coronally advanced flap technique. Based on clinical parameters, the authors concluded that despite both treatments leading to an effective reduction of periodontal-induced gingival recession, patients treated with both hyaluronic acid and the coronally advanced flap technique showed the best results. In particular, it has been demonstrated that the topical post-surgical application of hyaluronic acid was able to reduce the wound healing time, thus improving the wound stability and decreasing at the same time the infection risks [133]. 

The suitable application of hyaluronic acid in periodontal regeneration was further demonstrated by Babo et al. [134] through the synthesis of photo-crosslinkable hydrogel made up of methacrylated hyaluronic acid. This scaffold was realized in order to provide a suitable platform for the inclusion of platelet lysate, thus improving the in situ delivery of its derived growth factors and so promoting periodontal regeneration. Indeed, it is well known that platelet-lysate is a rich source of several growth factors, i.e., vascular endothelial growth factor, platelet derived growth factor, fibroblast growth factor (FGF), β1- and β2-transforming growth factors, epidermal growth factor, insulin-like growth factor and bone morphogenetic proteins. These latters strongly affect regenerative processes such as chemotaxis, cell proliferation/differentiation and the synthesis of extracellular matrix components i.e., fibrin, fibronectin and vitronectin, promoting the production of a suitable matrix to ensure the cell engraftment and migration. The realized hydrogels showed suitable elastic properties that improved by the increasing of platelet lysate amount, suggesting a proper performance of resulting scaffold during the mechanical stresses in the application site. The influence of this construct on cell growth was in vitro investigated on human Periodontal Ligament Fibroblasts (hPDLFs) cells, showing the best results for the hydrogel composed by the highest amount of platelet lysate (100% *v*/*v*). Moreover, the antibacterial effect of platelet lysate was tested on several microbial agents involved in periodontitis, demonstrating a high antimicrobial activity against methicillin resistant Staphylococcus aureus. These preliminary in vitro results strongly encourage the potential application of this scaffold as a delivery system for platelet lysate for both promoting periodontal regeneration and prevent/reduce the infections on injured sites [134].

Despite the numerous advantages of hyaluronic acid above described, its use in periodontal scaffold may be compromised by its poor physicochemical stability in the oral cavity, because of the presence of several chemical, physical and enzymatic stimuli [135]. For these reasons, usually this polymer is modified and/or associated with other materials in order to realize a suitable physical support for periodontal regeneration. An example of this approach was demonstrated by Miranda et al., who chemically modified hyaluronic acid and chitosan in order to promote cross-linking reaction, thereby stabilizing the resulting hydrogel, as above described [66].

#### 3.2.2. Collagen

Collagen is the most abundant protein in the body and the main component of the native extracellular matrix [48]. This protein is organized in fibrils (as quaternary structure) and to date 20 different kinds of collagen, with various functions according to the played role in the specific position, have been found in the body [136].

The most common one is collagen type I, mainly present in the dermis, bone, tendon, fasciae, sclera, organ capsules and fibrous cartilage. On the other hand, the type II of collagen is widely expressed in hyaline and elastic cartilage, while the type III of collagen is usually placed in the wall of the blood vessels, also such as copolymer with type I of collagen [124]. Due to its massive presence as a component of the extracellular matrix, currently the use of collagen in tissue regeneration field is widely investigated. 

However, this protein holds poor mechanical strength per se, thus making necessary the application of cross-linking processes in order to improve the stability of resulting con-structs.

Moreover, it is usually associated with other polymers, due to its rapid in vivo degradation through matrix metalloproteins, such as collagenases or serine proteases [137]. 

In these attempts, Rosdiani and co-workers [138] combined collagen, chitosan and glycerol (as plasticizer), realizing a scaffold with a reduced degradation rate in comparison with the one made up by only collagen, for a potential application in gingival recession therapy. The resulting construct showed suitable morphological and rheological properties with a pore range size between 102.4 and 143.5 μm, thus allowing an effective potential application in periodontal regeneration that requires a pore size ranging between 63 and 150 μm. The authors demonstrated the improved physicochemical proper-ties of resulting “hybrid scaffolds” showing their suitable swelling properties, that was directly proportional to the collagen content [138].

Another engaging application of collagen-based scaffold in periodontal regeneration was proposed by Kosen et al [139], through the realization of collagen hydro-gel/sponge construct. The resulting scaffold was in vivo tested on class II furcation defect in beagle dogs in order to evaluate its ability to properly guide the regeneration of damaged tissues. After two weeks from implantation, a new alveolar bone, periodontal ligament and cementum tissue was noticeable, becoming more evident after 4 weeks in comparison with negative control (animal group without scaffold implantation). Moreover, the scaffolds appeared quite stable, showing the first degradation products after 4 weeks. Therefore, the authors demonstrated an opportune use of this absorbable collagen-based hydrogel as physical support to improve the regeneration rate of periodontal tissues, showing a high in vivo tolerability with a low infiltration rate of inflammatory cells on it [139].

#### 3.2.3. Gelatine

Gelatine is a natural polymer obtained by the hydrolysis of collagen and shows a high biocompatibility, biodegradability and a good cost-benefit balance. Based on its production process, it can show two different forms: Type A and Type B, extracted by the collagen through a pre-treatment with acid or alkaline solution, respectively [140]. 

Recently, this biopolymer has been investigated for a potential use in periodontal regeneration. In these attempts, Ou et al. [141] realized a composed scaffold made up of zein/gelatine/hydroxyapatite in order to provide a physical support for human periodontal ligament stem cells (hPDLSCs). The features of this scaffold were compared to the ones owned by a similar scaffold realized without hydroxyapatite. In particular hydroxyapatite, because of its osteoconductive properties and high hydrophilicity, improved both adhesion and osteogenic differentiation of hPDLSCs seeded on it compared to the hydroxyapatite-free scaffold, in vitro. Moreover, the higher regenerative capability of zein/gelatine/hydroxyapatite embedding hPDLSCs in comparison with hydroxyapatite-free construct was also demonstrated in rats model, showing massive production of new bone tissue in animals in response to the presence of osteogenic material [141]. Therefore, this study emphasized the crucial significance to combine more materials in order to obtain an effective scaffold able to proper support periodontal regeneration.

A similar approach was proposed by Bottino et al. [142] through the realization of a multilayered scaffold. In particular, this construct was realized using a sequential multilayer electrospinning process and it is composed by a core-layer of poly-lactic-ε-caprolactone, PLA and gelatine coated with electrospun shall containing two functional materials, i.e., nano-hydroxyapatite (osteoconductive function) and metronidazole (antibiotic widely used in periodontitis). The resulting multilayered scaffold showed several advantages for a potential application in periodontal regeneration: high mechanical properties due to the presence of poly((lactic acid)-co-caprolactone) copolymer; suitable biocompatibility towards periodontal tissues due to the presence of gelatine, which mimics the components of the extracellular matrix, ensuring the adhesion, growth and differentiation processes of cells. Moreover, the incorporation of nano-hydroxyapatite and metronidazole allows to enhance osteoconductive behavior and prevent/reduce infections, respectively, affording the realization of a construct that reflects the complexity of periodontal tissues [142].

Another fascinating investigation concerning the use of gelatine was carried out by Sachar and co-workers [143], who realized a 3D nanofibrous gelatine scaffold in order to evaluate the matrix-cells and cells-cells interactions, in comparison with physiological environment. In these attempts, the authors have investigated the behavior of human gingival fibroblasts cells through confocal microscopy analysis, this type of cells maintains the integrity of oral and gingival tissues. The results showed that these fibro-blasts evenly migrated through the gelatine scaffold in the lower, upper and middle levels. Moreover, the allocation in this scaffold did not compromise the cell growth, showing a final cell number four-fold higher after 14 days than the one found on day 5. In addition, a noticeable extracellular matrix (mainly containing collagen type I) formation occurred after 14 days. This study confirmed that gelatine is a promising biomaterial for periodontal regeneration thanks to its ability to provide a useful environment for cells, thus allowing to promote the regeneration of oral and gingival tissues [143]. 

Moreover, the suitable use of gelatine-based scaffold for periodontal regeneration was further in vivo confirmed by Londero et al. [144]. The aim of this study was to realize a cheap scaffold (Gelfoam) to enhance the regenerative properties of loaded blood clot of dental tissue in immature teeth of dogs with pulp necrosis and apical periodontitis.

In particular, the association of Gelfoam and blood clot allowed a significantly higher formation of mineralized cementum-like tissue on dentinal walls and new functional tis-sues compared with use of blood clot alone [144].

The above-mentioned investigations focus the attention on the involvement of this biomaterial to realize an effective scaffold for periodontal regeneration, keeping the low production costs, thus suggesting a potential scalability from bench to chairside.

#### 3.2.4. Chitosan

Chitosan is a natural biopolymer derived from a partial deacetylation of the chitin. It is an important component used both in medicine and food fields [145,146]. Apart from the high mucosal biocompatibility and biodegradability, it is characterized by a hemostatic antimicrobial activity. Currently, it is one of the most used natural biomaterials and several studies concerning its applicability in the treatment of periodontitis and/or periodontal regeneration have been carried out during the last decade [147]. 

In these attempts, Varoni et al. [148] realized a tri-layered membrane to induce a simultaneous regeneration of hard and soft tissues that are normally compromised in the late stages of periodontitis. Starting from well-known bio-adhesiveness and wounds healing properties of chitosan, the authors crosslinked medium and low molecular weight chitosan and genipin obtaining a two-layered construct able to induce the regeneration of bone and gingiva. Moreover, a third layer of chitosan was deposited on it, in order to improve the regeneration of periodontal ligament. The cells (osteoblasts, gingival fibroblasts and human periodontal ligament fibroblasts) seeded on the resulting tri-layered scaffold showed a cell viability greater than 90% after 7 and 10 days, thus confirming its cyto-compatibility. These results were in vivo confirmed through periodontal ectopic model in mice, showing the absence of significant adverse reactions and a concomitant biodegradability [148].

The advantages of chitosan as biomaterial for the establishment of a useful scaffold in periodontal regeneration, were further demonstrated by Mota et al. [149]. In this study, the authors realized a chitosan-base composite scaffold embedding bioactive glass nanoparticles in order to promote bone and periodontal ligament regeneration. The effectiveness of the resulting construct was evaluated using human periodontal ligament cells (hPDL) and human bone marrow stromal cells (hBMSC). The results showed a reduced strength and elongation properties both in wet and dry conditions in response to the addition of bioactive glass nanoparticles to the scaffold. Conversely, in wet conditions, composite membrane resulted more flexible than nanoparticle-free scaffold, thus suggesting a suited mechanical feature for a potential in vivo application. Moreover, in vitro investigations showed higher metabolic activity of cells seeded on composite scaffold than ones on nanoparticles-free chitosan membrane after 28 days, especially hPDL, thus suggesting its potential use in periodontal regenerative medicine [149]. 

As well as for other described polymers, chitosan is often used in association with other materials in order to improve the performance of resulting scaffolds for both tissue regeneration and treatment of chronic diseases such as periodontitis. In this regard, Xuan et al. [150] synthesized a chitosan/ascorbate gel for the treatment of bacterial infections in periodontitis. Indeed, as widely described, the chronic inflammation of periodontium can lead to the destruction of periodontal supporting tissues, thus resulting in the loss of the entire dental apparatus. In particular, the purpose of this study was to evaluate the potential synergic antimicrobial effect of chitosan and ascorbate in the Wistar rats model. The effectiveness of chitosan/ascorbate gel was in vivo demonstrated by the reduction of several parameters, i.e., plaque index, gingival index, periodontal pocket depth, attachment level, and tooth mobility. The plaque index has been reduced in response to the electrostatic interaction between positive chitosan and negative dental plaque bacteria, thus leading to the inactivation of these latest. Moreover, because of its bioadhesive properties, chitosan resulted strongly associated to the root surface, thus improving the support of the dental structure and reducing at the same time the infiltration of inflammation-mediated cells. It provides a proper environment for periodontal regeneration. All these advantages have been accentuated by the association of the ascorbate [150]. 

#### 3.2.5. Fibrin

The fibrin is obtained by polymerization of fibrinogen catalyzed by thrombin in the last stage of the coagulation cascade [151]. In the few last decades, fibrin-based hydrogel was used for many regenerative applications, i.e., adipose, cardiovascular, ocular, muscle, liver, skin, cartilage and bone tissues engineering. It is physiologically present both in plasma and in the platelets α-granules and it plays an important role in platelets aggregation and haemostasis [152]. Fibrin-based scaffolds are usually realized starting from concentrated platelet-rich plasma (cPRP) as fibrin source [153]. In particular, fibrin is usually obtained from whole blood through sequential gradient centrifugations [154,155]. One of the main studied scaffolds realized with this natural polymer is hydrogel. Fibrin-based hydrogels show several suitable features: high biodegradability, non-immunogenicity and the ability to promote cell migration. On the other hand, its main disadvantages are the rapid hydrogel shrinkage after preparation and the poor mechanical strength. Based on this, usually this polymer is associated with other biomaterials such as fixing agents (poly-L-lysine) in order to improve mechanical stiffness properties of resulting scaffolds [156,157]. 

In these attempts, Li et al. [158] recently investigated the use of the Platelet-rich fibrin to realize a bioactive scaffold for the regeneration and repair of damaged periodontal tissues. The resulting scaffold was in vitro investigated by the evaluation of proliferation and migration rate of cells seeded on it (fibroblasts, dental follicle progenitors and alveolar bone osteoblasts), demonstrating a higher cellular activity compared to the same cell lines cultured in conventional plate. The suitable properties of this scaffold were also in vivo demonstrated, showing a complete integration of fibrin-base membrane after 3 months from implantation with new alveolar bone formation. Therefore, this study demonstrated the ability of platelet-rich fibrin membrane to both promote the regeneration of soft tissues and induce new bone formation, because of the presence of several growth factors delivered i.e., interwoven growth factors (such as TGF-β1, VEGF, IGFs, and PDGF-AB) and matricellular proteins (such as thrombospondin-1) [158]. 

Another study concerning the use of Platelet-rich fibrin (PRF) in periodontal regeneration was carried out by Duan et al. [159]. In particular, the authors focused their attention on the potential use of PRF-based membranes to promote regenerative properties of periodontal ligament stem cells (PDLSCs). This suggestion was previously in vitro con-firmed, showing an improved activity of alkaline phosphatase after 30 days of incubation and a high expression of osteogenic markers, i.e., OC, RUNX2 and BSP, which became noticeable after 21 days. In vivo results demonstrated a similar trend, showing the formation of new functional bone, cementum and periodontal fibers in mice, 24 days after the implantation of PRF-based membrane embedding PDLSCs. In particular, this construct provides the best results in terms of regenerative efficacy compared to cells-free collagen membrane, collagen membrane embedding cells and cells-free PRF-membrane. These results suggested that PRF has been the best choice as biological autologous material to realize periodontal scaffolds compared to collagen [159].

The advantageous use of fibrin-based scaffold as delivery system for stem cells in periodontal regeneration has been also demonstrated by Galler et al. [160]. Moreover, in order to reduce the rapid degradation of fibrin, polyethylene glycol (PEG) was added in the injectable gel composition. In this study, three types of stem cells were used: human exfoliated deciduous teeth cells (SHEDs) from the dental pulp of exfoliated incisors, dental pulp stem cells (DPSCs) from impacted wisdom teeth and periodontal ligament stem cells (PDLSCs), separated from the root surface of third molars after extraction. Bone marrow stromal stem cells (BMSSCs) were used as control. In vitro studies demonstrated that all testes cells proliferate up to 4 weeks, showing a high, intermediate and low alkaline phosphatase activity for PDLSCs, SHED/BMSSCs and DPSCs, respectively. Similar trend was also observed in terms of collagen production. Based on in vitro results, also considering the different proliferation rate of investigated cells, the authors used SHEDs for further in vivo investigations. Fibrin-based gel delivering the selected cells was then implanted in animal models. After 5 weeks from injection, the histological analysis demonstrated a complete degradation of fibrin that was replaced by new vascularized soft connective tissue. Therefore, the authors demonstrated both the higher capability of fibrin to support the regeneration of functional periodontal tissues and the easy applicability of realized injectable gel, thus emphasizing its potential use in humans [160].

Among the aforementioned natural materials, fibrin may provide an excellent accomplishment in periodontal tissue engineering, despite its relative novel use in this field. Indeed, it promotes the tissue regeneration by itself because of the large amount of embedded growth factors and the large availability in the blood, thus cutting down the production costs.

Furthermore, based on critical reviews reported in literature, it is our opinion that the association of natural and synthetic materials could provide the best goal in this field, taking into account the main differences between the two classes of materials (Table 1).

## 4. Composite Scaffolds

Periodontitis is strongly associated with severe infections and chronic inflammation that, during the advanced stages, may compromise the supporting tissues, leading to the total loss of teeth [161]. In these attempts, regenerative approaches are usually associated with antimicrobial/anti-inflammatory chronic therapies [162]. In order to improve the efficacy of these latest, reducing at the same time their systemic side effects, several investigations have been focused on the use of therapeutic nanosystems directly administered in situ [163].

Indeed, nanosystems, i.e., polymeric nanoparticles and nanovesicles, have shown the ability to increase the efficacy of payloads, providing their sustained and controlled release, in comparison with the same drugs administered in the free form [164].

**Figure 3:**
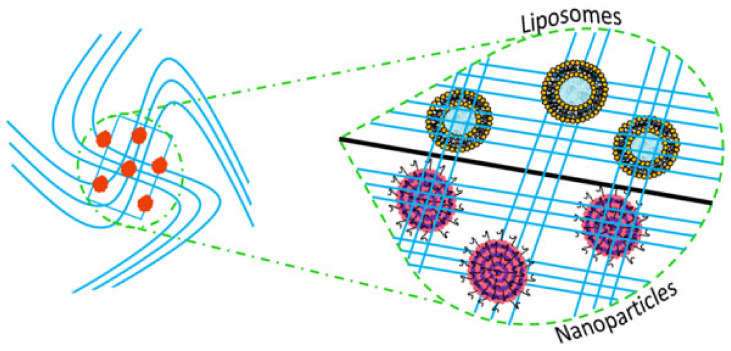
Schematic representation of composite scaffolds, made up of nanosystems embedded into three-dimensional (3D) scaffold.

In these attempts, a promising approach in periodontal diseases may be represented by the embedding of therapeutic nanosystems into 3D scaffolds in order to reduce their systemic reuptake and provide a reservoir “double matrix” [165,166] (Figure 3).

This approach may reduce several drawbacks associated with conventional oral therapies, such as limited gastro-intestinal absorption, first-passage liver effect, rapid inactivation and clearance of drugs, thus improving the patients’ compliance in response to reduced administration frequency and lower side effects [167,168]. Furthermore, these nanosystems have been proposed also for growth factors delivery, i.e., osteogenic factors as bone-morphogenetic protein-2, showing an improved behavior and biological effect than the ones administered in the free form [169].

Despite the above-mentioned fascinating advantages of this potential approach, several efforts are still required to realize an effective “composite scaffold.” In particular, the main challenges are the hardness to control exactly the physicochemical properties of the embedded nanosystems during all realization stages and not least the high cost of production [170].

Below, we mention the most used nanosystems and the current development of this innovative perspective.

### 4.1. Liposomes

Liposomes, the most successful drug delivery systems to date, were described for the first time by Bangham et al. [171]. These nanometric vesicles show a spherical-like structure and are composed by the combination of natural and/or synthetic phospholipids, cholesterol and surfactants, making the bilayer and a certain volume of aqueous phase in the core [172]. Through their amphiphilic compositions, liposomes are able to encapsulate hydrophilic, amphiphilic and hydrophobic bioactives in their aqueous core, at the bilayer-aqueous core interface or in lipid compartment, respectively [173]

Liposomes are biocompatible, biodegradable and, above all, they are characterized by an accentuated formulation and application versatility [174,175]. They are useful in several fields i.e., cosmetics, food and pharmaceutical [176]. About this latter, nanovesicles can be administered through different routes i.e., systemic, ocular, transdermal ones and others [177,178,179]. Moreover, thanks to their above-mentioned high versatility, liposomes lend themselves to structural modifications, allowing an improved behavior, such as increased half-life and shelf-life in response to the surface modifications (such as PEGylation) [180], a site-specific delivery of payloads in response to their surface functionalization (such as immunoliposomes) [181] or a higher loading capability (such as cationic liposomes for gene delivery) [182].

Despite their application in several pathologies, today only few investigations in literature describe the liposomes application in periodontal affections. 

An example concerning the involvement of liposomes in periodontal diseases was carried out by Hu et al. [183]. In this study, the authors focused their attention on the possibility to exploit the decreased pH of the oral cavity in response to the bacterial-mediated sucrose fermentation. Indeed, this latter leads to the formation of plaque, creating an acid environment suitable for the growth of pathological microorganisms. Starting from this, a doxycycline-loaded pH-sensitive liposome was realized and investigated for potential treatment. The resulting nanosystems demonstrated a site-specific release of payload, due the structural destabilization induced by pH value below than 5. The resulting pH-sensitive therapeutic liposomes showed a suitable bacteriostatic effect on tested bacteria (*P. gingivalis* and *P. intermedia* strains), leading to an inhibition rate (around 75%). Moreover, the antimicrobial effect of the liposomes was further increased by the chitosan-based coating. In detail, the authors used a chitosan modified with quaternary ammonium groups, which provided a higher positive charge coating on liposomes surface. The modified chitosan coating allowed to increase the antibacterial effect of resulting nano-vesicles thanks to the electrostatic interaction between cationic coating and bacterial membranes. Therefore, this approach demonstrated the possibility to improve the efficacy of periodontal treatment by exploiting the intrinsic microbial characteristics and the stimuli provided by their metabolic activity, i.e., the affinity of microbial membrane surface for chitosan-coating and the resulting decreased pH in the plaque, respectively [183].

Another example of modified liposomes for periodontal application was explored by Di Turi et al. [184]. In this investigation, magnetic liposomes were used to better control their diffusion into the dentinal tubules, through the application of an external magnetic-drive force. In particular, the magnetic responsiveness of resulting nano-vesicles was provided by the inclusion of magnetite nanoparticles into the PEGylated liposomes core. This approach, associated with their nanometric size (d < 300 nm), led to a more rapid movement of liposomes within the tubules, allowing their penetration in deeper terminal sites of tubules. This approach could result in useful in situ treatment of periodontitis. In fact, the loading of drugs in investigated magnetic nanovesicles may permit to overcome the poor penetration rate of conventional nanovesicles in deeper regions of periodontal pocket, thus resulting in an improved therapeutic efficacy [184]. 

Due to the different membrane receptors expressed by microorganisms in comparison to the human cells, a potential approach to treat periodontal diseases could be represented by the use of immunoliposomes, delivering antimicrobial agents. Based on this, Robinson et al. [185] realized immunoliposomes with surface-bound monoclonal antibodies, able to recognize antigens expressed on the surface of *S. oralis*. Despite the lower specificity of anti-oralis immunoliposomes compared to free antibody against investigated microorganism, due to the higher steric hindrance of liposomes and its interactions with other microorganisms in the oral cavity, the authors demonstrated a proper targeting ability toward *S. oralis*. Moreover, the effective targeting did not depend on the used lipid mixture, because of the ability of conjugated antibody molecules on liposomal surface to mask the net charge of native bilayer, highlighting a suitable formulation versatility [185].

Despite the advantages above described, the in situ administration of liposomes per se may result in difficulty and require the use of high-potency drugs, in response to the relatively small area of application site. To overcome these restrictions, a potential strategy could be represented by their inclusion into 3D scaffolds, thus allowing the formation of “composite scaffold.” Indeed, this fascinating strategy may improve the residence times of embedded nanosystems in the periodontal tissues, allowing a slower and sustained release of carried payloads. Unfortunately, to the best of our knowledge, to date no investigations involving the liposomes’ inclusion in a specific scaffold for periodontal application have been carried out.

### 4.2. Polymeric Nanoparticles

Nanoparticles are colloidal nanosized carriers able to reach the deepest tissue layers in response to their small mean size They show several advantages mainly about the ability to increase the payloads’ stability, providing at the same time a controlled and sustained release [167]. Different studies were carried out to evaluate their efficacy where the small size could play a key role for an easier accommodation in the periodontium than micrometric particles [186]. 

On these bases, Lee et al. [187] realized PLGA/chitosan-based nanoparticles in order to provide a proper nanoplatform for the local delivery of two drugs with different pharmacological effects: lovastatin and tetracycline. The aim of this study was to obtain a controlled and site-specific release of drugs to simultaneously reduce periodontal infections and promote tissue regeneration. The resulting nanocarriers demonstrated a burst release of tetracycline on day 1 followed by a sustained release until day 14. On the other hand, lovastatin showed a slower release than tetracycline up to day 21, thus allowing a proper condition to provide a suitable tissue regeneration. The antibacterial activity of resulting double-loaded nanoparticles was in vitro investigated, showing a clear inhibition zone of tested *Aggregatibacter actinomycetemcomitans* and *Prevotella ingrescens* for the entire work (7 days). Conversely, the regenerative properties were evaluated in periodontal defects of dogs, demonstrating both a higher new bone formation and improved deposits of cementum on the root surface than control groups (not treated), after administration of nanoparticles as a hydrogel prepared by mixing them with gelatine [187]. 

Alternative to the lipid-based nanovesicles, recently some investigations concerning the use of polymeric nanoparticles embedded in 3D nanosystems for a potential application in periodontal tissue regeneration, have been explored.

In these attempts, Lee et al. [188] realized a chitosan/polyurethane nanofiber membrane loading silver nanoparticle in order to provide a composite scaffold with anti-microbial and regenerative effects. The cytocompatibility of the resulting construct was in vitro investigated on NIH 3T3 cells, demonstrating a suitable cell viability up to 48 h (higher than 80%) without significant differences compared to the nanoparticle-free scaffold. These results suggested that the inclusion of silver nanoparticles in the studied scaffold did not compromise its biological compatibility. Moreover, its antimicrobial features were tested on *Porphyromonas gingivalis*, a key mediator of periodontitis, showing a suitable inhibition of bacterial proliferation in a silver concentration-dependent manner. These preliminary results highlight the potential use of metal nanoparticles to manage periodontitis and its potential application in periodontal tissues through their inclusion in hybrid membrane [188].

Another thrilling approach combining the advantages of 3D scaffold and nanosystems in periodontal affections was described by Shen et al. [189]. In this study, the authors investigated the effectiveness of chitosan-based nanoparticles embedding into PLA nanofibers’ scaffold. The presence of chitosan nanoparticles improves the mechanical properties of composite supports compared to nanoparticles free ones. Moreover, the bagnability increased in response to chitosan nanoparticles presence, due to the high hydrophilicity of this polymer, thus providing better cell attachment. The chitosan also improved mechanical strength of the fibers. Furthermore, after an optimization of the composite construct, the authors investigated its ability to provide a proper support for cells growth and differentiation, taking into account two different cell lines: BMSC (bone marrow stem cells) and hPDLCs (human periodontal ligament cells). Both cell lines demonstrated a significant growth in the first three days of treatment. Finally, BMSC showed the presence of mineralized tissue after 2 weeks, in particular in the treatment with composite scaffold compared to the nanoparticles free one [189].

One of the main common consequences of periodontitis is the increased production of gingival crevicular fluid (a fluid secreted by gums), which invalidates or strongly reduces the anti-inflammation therapies’ efficacy, enhancing the clearance of bioactives from the injured site [190]. One of the main useful approaches to overcome this drawback is the use of mucoadhesive gels that provide a localized therapy, thus both improving the treatment efficiency and reducing the administration rate [191]. 

In order to improve the efficiency of this strategy, recently Singh et al. [192] realized a mucoadhesive gel containing ganglioside-coated polycaprolactone satranidazole- loaded nanoparticles. In particular, satranidazole (a potent antibiotic drug) was delivered through polycaprolactone nanoparticles in order to prevent its potential interference with gel structure and to reduce its marked hydrophobicity. On the other hand, ganglioside (0.1% *w*/*v*) was used as coating material to circumvent the immune response after implantation. The resulting nanoparticles were loaded into sodium carboxymethyl cellulose gel and its features were compared with free-nanoparticle gel with the same composition. The analysis of mechanical parameters did not demonstrate any significant changes in response to the addition of nanosystems. Moreover, in vitro evaluation on *A. actinomycetemcomitans* showed a higher antibacterial activity of composed scaffold compared to the free drugs and the empty gel, thus confirming its optimal features. Based on the promising effects in vitro, a clinical study has been carried out enrolling 10 patients with chronic periodontitis. Both nanoparticle-loaded scaffold and free-nanoparticles one were implanted and their effectiveness were evaluated in terms of probing depth (PD), plaque index (PI) and gingival index (GI). In particular, the results demonstrated a higher antibacterial activity of the gel containing nanoparticles, showing also a suitable infection management, thus preventing the formation of new lesions. Probably it was due to the ability of nanocarriers to provide a sustained drug release, acting as a reservoir system. Furthermore, no significant side effects were detected, confirming the biocompatibility of the composite construct investigated. Therefore, these results emphasized a useful application of proposed approach, also due to the crucial presence of ganglioside, which reduces the immunological response and prevents the inflammation-mediated destruction of periodontal tissues, thus reducing remission time [192].

The studies above-reported demonstrate the great potentials of a periodontal treatment that combines the nanometric systems’ and scaffolds’ advantages. However, this approach is still in the beginning stage; thus, further investigations need to be required in order to provide an effective periodontal tissue regeneration.

## 5. Conclusions and Future Perspectives

During the last few years, the researchers took important advancements in the regeneration of periodontal tissues, demonstrating the key role of scaffolds in the managing tissue renewal and repair. In particular, the choice of appropriate materials with suitable bio-physical and mechanical features appears to be a crucial point. In these attempts, in the authors’ opinion, natural materials physiologically present in the extracellular matrix, i.e., collagen, gelatine and particularly fibrin, may provide a proper starting point to realize 3D scaffold mimicking the physiological environment in periodontium. Unfortunately, usually this bio-polymer shows poor physicochemical and enzymatic stability, thus requiring chemical modification and/or the association of synthetic material in order to improve the features of resulting constructs.

Despite its relatively novel application in periodontal regeneration, one of the main promising approaches to date concerns the combination of 3D scaffold and nanosystems. Indeed, this strategy could lead to an improved effectiveness of resulting “composite scaffolds,” providing at the same time physical support for tissue regeneration and a better controlled release of payloads i.e., anti-inflammatory drugs and/or growth factors. On the other hand, one of the main restrictions is the higher costs of production that are associated with the limits of current technologies available, reducing its clinical appeals.

Therefore, despite these fascinating suggestions, further efforts are required in order to provide a real effectiveness platform that could justify the improved cost of care, hence speeding up the translation of this technology from the laboratory to the clinic.

## Figures and Tables

**Table 1:** Main differences between natural and synthetic materials used in periodontal regeneration.

Synthetic Materials	Natural Materials
Produced through high controlled processes and on large scale	Obtained from different renewable sources
No variation batch to batch	Variation batch to batch
Easy manipulation and purification	Arduous purification processes
High mechanical properties	Poor mechanical properties
Reproducible degradation processes/rate	Unpredictable degradation rate
Low bioactivity and poor cellular adhesion/growth	Biomimetic surface and/or natural remodelling
